# Furuncular Myiasis Mimicking an Epidermal Inclusion Cyst of the Scalp: A Case Report and Focused Review of Diagnostic Pitfalls in Scalp Myiasis

**DOI:** 10.7759/cureus.112656

**Published:** 2026-07-14

**Authors:** Sri Naidnur, Kasey Smith, Kara Asbury, Bryan Gammon, Rick Lin

**Affiliations:** 1 Dermatology, Lake Erie College of Osteopathic Medicine, Greensburg, USA; 2 Dermatology, Oasis Dermatology Group, McAllen, USA; 3 Dermatology, Hospital Corporation of America (HCA) Healthcare, Bradenton, USA; 4 Dermatology, Hospital Corporation of America (HCA) Healthcare Corpus Christi Medical Center Bay Area Program, McAllen, USA; 5 Dermatopathology, Sagis Diagnostics, Houston, USA

**Keywords:** costa rica, cyst, furuncular myiasis, myiasis infestation, scalp myiasis

## Abstract

Furuncular myiasis of the scalp may closely mimic common cystic scalp lesions, including pilar and epidermal inclusion cysts, leading to delayed diagnosis and inappropriate treatment. We report the case of a 67-year-old man who presented with a painful right parietal scalp nodule that was initially diagnosed as a pilar cyst or epidermal inclusion cyst. Mild discharge, erythema, and inflammation partially improved following a two-week course of oral doxycycline; however, persistent pain prompted the patient to pursue surgical excision. Histopathologic examination revealed findings consistent with furuncular myiasis. At postoperative follow-up, additional history revealed recent travel to Costa Rica with outdoor exposure, including hiking and ATV riding.

To better characterize diagnostic pitfalls associated with scalp myiasis, we conducted a focused review of published cases, with an emphasis on initial misdiagnoses, diagnostic methods, and management approaches. Reported cases were mistaken for sebaceous cysts, epidermal inclusion cysts, furunculosis, abscesses, cellulitis, infected insect bites, and, less commonly, osteomyelitis. Diagnosis was established through several approaches, including clinical recognition, ultrasound, histopathologic examination, the direct visualization of larvae, and the reconsideration of the clinical history. We summarize selected published cases, along with their initial diagnoses, diagnostic methods, and management strategies, in a table to highlight recurring diagnostic pitfalls.

This case report and focused review demonstrate that scalp myiasis remains an important mimic of common cystic scalp lesions. Clinicians should consider myiasis in patients with painful scalp nodules, central puncta, incomplete response to antibiotic therapy, and relevant travel exposure. Although the lesion in our patient was clinically favored to represent a benign cystic process, definitive diagnosis was not possible on examination alone. Excision and subsequent histopathologic evaluation established an unexpected diagnosis and facilitated appropriate management.

## Introduction

Cutaneous myiasis is a parasitic infestation caused by the larvae of various fly species and includes furuncular, migratory or creeping, and wound or traumatic forms [1,2]. Furuncular myiasis is the most common presentation in travelers returning from Central and South America and is most frequently caused by *Dermatobia hominis* [1]. Patients typically present with a painful nodule that may have a central punctum and discharge [1,2].

When these lesions occur on the scalp, diagnosis can be challenging because they often resemble much more common conditions such as pilar cysts, epidermal inclusion cysts, furunculosis, and abscesses [1,2]. As a result, patients may receive antibiotics, undergo imaging studies, or undergo surgical excision before the correct diagnosis is made. Several published reports have described delayed recognition, with the diagnosis ultimately established through travel history, imaging, histopathologic evaluation, direct visualization of larvae, or larval extraction [3-13].

We present a case of furuncular myiasis of the scalp initially diagnosed as a pilar cyst or epidermal inclusion cyst. We also review selected published cases of scalp myiasis, with an emphasis on initial misdiagnoses, diagnostic methods, and management approaches, to highlight recurring diagnostic pitfalls and practical clinical lessons.

## Case presentation

A 67-year-old man presented to the dermatology clinic with a painful nodule on the right parietal scalp that had developed approximately seven weeks earlier (Figure 1A). Physical examination demonstrated a tender subcutaneous nodule on the scalp with a central punctum and discharge; based on the anatomic location and cystic clinical appearance, the differential diagnosis included an inflamed pilar cyst or an epidermal inclusion cyst (Figure 1B). The exact lesion diameter was not recorded; however, the excision ellipse measured 1.3 × 2.2 cm. The patient denied a sensation of movement within the lesion and did not report fever or systemic symptoms. Laboratory studies, dermoscopy, and ultrasound were not performed prior to excision because the lesion was clinically favored to be cystic.

**Figure 1:**
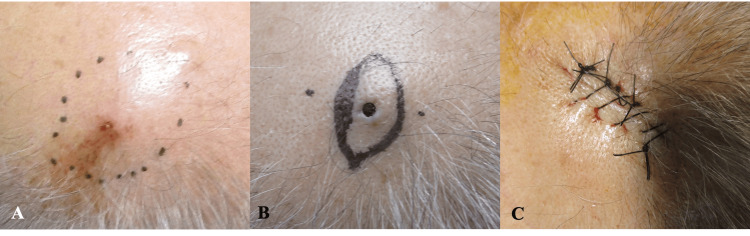
Clinical images of the right parietal scalp lesion. (A) Painful scalp nodule with a central draining punctum and surrounding inflammation; the dotted line marks the lesion boundaries approximately seven weeks after onset. (B) Dome-shaped scalp nodule with a central punctum, marked for subsequent excision. (C) Appearance after excision and primary closure approximately 10 weeks after onset.

The surrounding erythema and inflammation partially improved following a two-week course of oral doxycycline. Because the lesion remained painful and caused persistent discomfort, the patient elected to proceed with surgical excision, which was performed approximately 10 weeks after lesion onset (Figure 1C).

Histopathologic examination revealed a cystic cavity with mixed inflammation and an organism with an undulating chitinous exoskeleton and pigmented spines, findings consistent with myiasis (Figure 2). The larva was removed from within the excised specimen and identified histopathologically.

**Figure 2:**
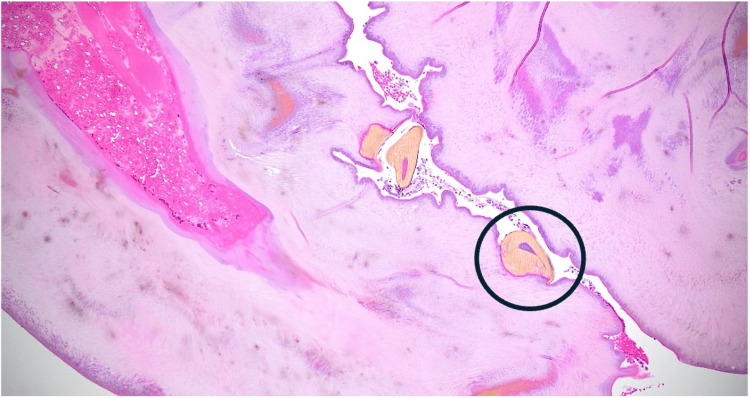
Histopathologic findings of furuncular myiasis. Hematoxylin and eosin stain, ×100 magnification, demonstrating larval cuticular structures with pigmented spines within the lesion cavity (black circle).

At a postoperative follow-up visit approximately three weeks later for suture removal, the pathology results were reviewed, and additional history was obtained. The patient reported that the lesion developed shortly after returning from a one-week trip to Costa Rica, during which he participated in outdoor activities, including hiking and ATV riding with significant mud exposure. Based on the clinical presentation, travel history, and histopathologic findings, *Dermatobia hominis* was considered the most likely causative organism.

## Discussion

A review of published cases of scalp myiasis identified recurrent patterns of delayed recognition and initial misdiagnosis [3-13]. This was conducted as a focused review of published English-language cases of scalp myiasis identified through targeted online literature searches and reference screening of retrieved articles. Search terms included “scalp myiasis,” “furuncular myiasis scalp,” “cutaneous myiasis scalp,” and “*Dermatobia hominis* scalp.” Cases were included when scalp involvement was described and when the initial diagnosis, diagnostic method, or management approach was documented. Initial diagnoses included sebaceous cysts, furunculosis, abscesses, cellulitis, infected insect bites, bacterial superinfection, and, less commonly, suspected calvarial osteomyelitis [3-13]. Across the reviewed cases, diagnosis was ultimately established through clinical recognition, imaging, histopathologic examination, the direct visualization of larvae, intraoperative discovery, or the reconsideration of the clinical history [3-13]. These cases are summarized in Table 1.

**Table 1:** Selected scalp myiasis cases with documented misdiagnosis or delayed recognition. Cases are arranged with the present case first, followed by published cases in reverse chronological order. The Al-Dabbagh and Douri case was acquired locally without international travel and is included to illustrate the range of diagnostic presentations [12]. NR, not reported or not retrievable from accessible source material; M, male; F, female

Age/Sex (Reference)	Initial Diagnosis/Differential	Diagnostic Method	Management
67/M (present case)	Pilar cyst versus epidermal inclusion cyst	Histopathology after excision; travel history obtained afterward	Surgical excision
11/M [13]	Suspected staphylococcal infection	Ultrasound	Larval removal
21/F [12]	Furunculosis	Direct visualization of larvae	Larval extraction
50/F [8]	Cystic scalp lesion/abscess	Ultrasound	Larval removal
42/M [6]	Infected sebaceous cyst	Clinical recognition following travel history	Larval extraction
62/M [4]	Sebaceous cyst, furunculosis, insect bite, cellulitis, and leishmaniasis	Histopathology after excision	Surgical excision
3/M [9]	Scalp abscess; concern for calvarial osteomyelitis	MRI excluded abscess/osteomyelitis; laboratory identification after extraction	Surgical incision
12/F [10]	Infected psoriatic scalp lesions/bacterial superinfection	Direct visualization of larvae	Occlusion and manual extraction
50/M [7]	Cellulitis and an infected insect bite	Clinical recognition following travel history	Larval extraction
NR [11]	Furunculosis, abscess, and insect bite reaction	Clinical recognition	Larval extraction
39/F [3]	Sebaceous cyst	Histopathology after excision	Surgical excision
NR [5]	Infected sebaceous cyst	Intraoperative discovery of larvae	Surgical excision

The most striking finding from the reviewed cases was the frequency with which scalp myiasis was initially mistaken for common scalp lesions [3-13]. Cyst mimicry emerged as one of the most consistent themes identified in the literature [3-6]. Our patient was initially diagnosed with a pilar cyst or epidermal inclusion cyst and underwent surgical excision before the diagnosis was established. Dome-shaped nodules, discharge, tenderness, and a central punctum may closely resemble inflamed cysts or other common scalp lesions [1,2].

Another recurring finding was initial treatment or diagnostic framing as a bacterial process [6-8,10,12,13]. Antibiotic therapy was reported in several reviewed cases before the diagnosis of myiasis was established [10,12,13]. Although antibiotics do not eradicate larvae, improvement in surrounding inflammation may reinforce an incorrect diagnosis and delay further evaluation [1,2]. In our patient, discharge, erythema, and inflammation improved following doxycycline therapy, but persistent tenderness and discomfort led the patient to elect surgical excision.

Travel history emerged as a particularly important clue in cases of travel-associated furuncular scalp myiasis [3,6-9,13]. In several reviewed cases, including the present case, travel history was obtained only after the diagnosis was suspected or established [3,6]. Although travel history is not diagnostic on its own, the recognition of travel to endemic regions may substantially narrow the differential diagnosis and prompt the earlier consideration of myiasis [1,2].

Histopathologic examination served as the primary diagnostic modality in our case and played a critical role in several published scalp cases [3,4,9]. In multiple reviewed cases, diagnosis was established only after excision or procedural removal, followed by the pathologic evaluation of a presumed cystic or inflammatory lesion [3,4,9]. Although the lesion was clinically favored to represent a benign cystic process and no features specifically suggested malignancy, definitive diagnosis was not possible on examination alone. Excision and subsequent histopathologic evaluation established an unexpected diagnosis that would not have been readily suspected clinically.

Management varied depending on when the diagnosis was recognized [1,2]. Once myiasis was suspected, many patients were successfully treated with larval extraction, occlusive techniques, or limited procedures to remove the organism [1,2]. In contrast, patients whose lesions were presumed to represent cysts, abscesses, or other scalp pathology often underwent surgical excision or procedural removal before the diagnosis was established [3-9]. Despite differences in management, outcomes were generally favorable once the larva was removed [1,2].

This focused review was limited to published English-language cases and may be subject to publication bias. When myiasis is suspected, ultrasound may support noninvasive diagnosis [8,13], and occlusive techniques may be considered before surgical intervention [1,2]; histopathologic examination remains valuable when the diagnosis is made after excision [3,4,9].

The reviewed cases suggest that scalp myiasis is often recognized after persistent symptoms prompt further evaluation, including imaging, histopathologic examination, direct visualization of larvae, or reconsideration of the clinical history [3-13]. Maintaining suspicion for myiasis in patients presenting with painful scalp nodules, central puncta, incomplete response to antibiotic therapy, or relevant travel exposure may help reduce diagnostic delay and unnecessary interventions.

## Conclusions

Scalp myiasis remains an uncommon but important mimic of common cystic and infectious scalp lesions. Our case report and focused review demonstrate that delayed recognition is common, with many reported cases initially diagnosed as cysts, furunculosis, abscesses, cellulitis, or infected insect bites. Recognizing the scalp as a possible site of myiasis and obtaining a detailed travel history when it is suspected may broaden the differential diagnosis and facilitate earlier recognition. The awareness of these recurring diagnostic pitfalls may help reduce unnecessary interventions and improve patient care.
